# Current and Future Therapies for Multiple Sclerosis

**DOI:** 10.1155/2013/249101

**Published:** 2013-02-07

**Authors:** Alireza Minagar

**Affiliations:** Department of Neurology, Louisiana State University Health Sciences Center, 1501 Kings Highway, Shreveport, LA 71130, USA

## Abstract

With the introduction of interferon-**β**1b in 1993 as the first FDA-approved treatment for multiple sclerosis, the era of treatment of this incurable disease began, and its natural course was permanently changed. Currently, seven different treatments for patients with multiple sclerosis with different mechanisms of action and dissimilar side effect profiles exist. These medications include interferon-**β**1a intramuscular (Avonex), interferon-**β**1a subcutaneous (Rebif), interferon-**β**1b subcutaneous (Betaseron/Extavia), glatiramer acetate (Copaxone), natalizumab (Tysabri), fingolimod (Gilenya), teriflunomide (Aubagio), and mitoxantrone (Novantrone). In addition, a large number of clinical trials are being conducted to assess the safety and efficacy of various experimental agents in patients with multiple sclerosis, including alemtuzumab, dimethyl fumarate, laquinimod, rituximab, daclizumab, and cladribine. In this paper, the author presents a concise and comprehensive review of present and potential treatments for this incurable disease.

## 1. Introduction

Multiple sclerosis (MS) is an immune-mediated demyelinating disease of the human central nervous system (CNS), which causes neurological disability in young adults [1, 2]. Presently, the etiology and cure for MS remain unknown. MS develops in individuals who are genetically susceptible following exposure to an unidentified environmental agent. Extensive epidemiological studies indicate that genetic factors play a significant role in the development of MS [1, 3, 4]. In addition, certain environmental factors such as exposure to or infection with certain viruses such as Epstein-Barr virus, low serum vitamin D levels, and smoking may contribute to the development of MS.

MS is commonly a disabling disease and remains the leading cause of acquired neurological disability in young adults individuals between 15 to 45 years [5]. The peak age of onset is 29 years, and similar to other immune-mediated diseases, in most forms of MS, females outnumber the males. MS affects both white and gray matters of the CNS (whole brain disease), and its underlying neuropathology leads to loss of myelin/oligodendrocyte complex as well as neuronal and axonal degeneration (demyelination versus neurodegeneration) [6]. 

Clinically, MS presents with four relatively distinguishable patters: relapsing remitting (RRMS), secondary progressive (SPMS), primary progressive (PPMS), and relapsing progressive (RPMS). The most common clinical phenotype of MS, RRMS, may initiate with a single uni- or multifocal demyelinating attack, referred to as clinically isolated syndrome (CIS) [7]. This first attack is usually followed by similar or different forms of relapses in time. Patients may recover from these attacks completely or partially, with or without treatment. The majority of MS patients initially present with relapsing-remitting form of MS, which is characterized by more neuroinflammation than neurodegeneration [8]. This concept clinically manifests with relapses and formation of new MRI lesions, particularly contrast-enhancing T1-weighted lesions. Over the course of years, each relapse may leave the patient with some residual disability which slowly accumulates, leading to permanent disability. Within 10 years from the initial diagnosis, most patients with RRMS enter the secondary progressive (SPMS) form of MS, which presents with less inflammation and more neurodegeneration [9]. Clinically, patients with SPMS present with progression of neurological disability. Primary progressive MS is the least frequent form of MS and is recognized by the absence of the relapses and progressive deterioration of neurological status from the onset, scarcity of the neuroinflammation within the CNS, and paucity of clinical relapses [9, 10]. Patients with PPMS usually do not demonstrate significant recovery from the progressive disease. Unlike patients with RRMS, male and female individuals are equally affected by PPMS. 

Currently, MS remains an incurable condition. However, a number of treatments with varying efficacy and adverse effect profiles have been approved by the FDA. These medications include beta-interferons, glatiramer acetate, Tysabri, Gilenya, and mitoxantrone. Very recently, a new disease modifying agent, teriflunomide (Aubagio), was approved by the FDA for treatment of relapsing forms of MS.

## 2. Beta-Interferons

Beta-interferons which include two forms of interferon-*β*1a (IFN-*β*1a) and interferon-*β*1b [IFN-*β*1b], are type 1 interferons and approved by the FDA for treatment of MS and CIS. Three different formulations of beta-interferons which exist for treatment of MS consist of low dose IFN-*β*1a (Avonex) (30 micrograms intramuscular once weekly), high disease IFN-*β*1a [Rebif] (22 and 44 micrograms subcutaneously three times weekly), and IFN-*β*1b (Betaseron) (8,000,000 units 250 micrograms subcutaneously alternate day injection). Due to the difference in frequency of administration and the utilized dose, Avonex is known as “low dose” *β*-interferon, while Rebif and Betaseron are recognized as “high dose.” These three beta-interferon formulations along with glatiramer acetate are recognized as first-line disease modifying agents for treatment of MS [11]. The mechanisms of action of beta-interferons are discussed, and then their clinical trials and side effects will be presented in the order of their approval in the US.

## 3. Beta-Interferons: Mechanisms of Action

While detailed mechanism(s) of the therapeutic actions of the *β*-interferons remains incompletely understood, their beneficial impact in MS patients may stem from their anti-inflammatory properties as well as their effects on the endothelial cells of the blood brain barrier. It has been demonstrated that IFN-*β* decreases antigen presentation [12], has potential modulatory effects on costimulatory molecules present on dendritic and other cells [13, 14], suppresses proliferation of the Th1 cells and increases expression of IL-10 (a major anti-inflammatory cytokine) [15], and shifts the inflammatory environment from proinflammatory to anti-inflammatory [16, 17]. IFN-*β* as a class does impact the endothelial cells and block the disintegrating effects of the IFN-*γ* on cerebral endothelial cells [18], decrease plasma endothelial microparticles which act as promoters of transendothelial migration of the activated leukocytes [19], protect endothelial cells from apoptosis induced by serum from MS patients [20], and decrease the expression of matrix metalloproteinases, which participate in disruption of the subendothelial matrix [21, 22].

## 4. Beta-Interferons: Clinical Trials

In 1993, IFN-*β*1b (Betaseron/Extavia) was the first immunomodulatory agent approved by the FDA for the treatment of patients with RRMS [23, 24], and it is also approved for treatment of patients with SPMS who still experience relapses [25]. Utilizing recombinant DNA technology, IFN-*β*1b is produced by *Escherichia coli* [26]. The dosage of IFN-*β*1b is 250 *μ*g subcutaneously every other day. 

A number of clinical trials have assessed the efficacy and safety of IFN-*β*1b in patients with MS [23, 24]. In 1993, during a phase 3 double-blind, placebo-controlled clinical trial, IFN-*β*1b was evaluated in a cohort of 372 patients with MS having an EDSS score of 0.0–5.5 and who had experienced at least two relapses in the two years prior to study initiation. Study subjects were randomly treated with placebo or IFN-*β*1b (500 or 250 *μ*g subcutaneously once every other day) for 24 months. Based on the results of this initial study, utilization of IFN-*β*1b in MS reduced clinical relapse rate, in both treatment groups, compared to the placebo (higher dose versus placebo *P* = 0.0001; lower dose compared to placebo *P* = 0.01). In addition, patients who were treated with the higher dose of IFN-*β*1b compared to those treated with the lower dose showed more decrease in their clinical relapse rate (*P* = 0.0086), which in turn indicated a dose effect. MR neuroimaging results also revealed reduction of T2-weighted active lesions (higher dose IFN-*β*1b compared to placebo, *P* = 0.0089; lower dose IFN-*β*1b compared to placebo, *P* = 0.04). The number of new T2-weighted lesions decreased (higher dose IFN-*β*1b versus placebo, *P* = 0.0026; lower dose compared to placebo, *P* = 0.03) so did the MRI burden of the disease (higher dose IFN-*β*1b compared to placebo, *P* < 0.001; lower dose compared to the placebo, *P* = 0.04). During this trial, treatment of MS patients with IFN-*β*1b did not show any superior effect over placebo on progression of disability. 

Another study addressed the long-term safety and efficacy of IFN-*β*1b in the treatment of MS patients with RRMS. During this multicenter, open-label, observational study which was conducted for up to 16 years, cross-sectional data from the participants in the pivotal trial of IFN-*β*1b was utilized [27]. The findings of this study indicated that early and uninterrupted long-term therapy of MS patients with IFN-*β*1b was acceptable since the decrease in the relapse rate stayed similar to the initial study. In addition, longer period treatment of patients with RRMS with IFN-*β*1b was associated with slowing of progression of disability [28]. 

 Frequent side effects of IFN-*β*1b consist of flu-like symptoms, headache, injection site reactions, asthenia, lymphopenia, elevated hepatic enzymes, and pain. Less commonly encountered but more serious adverse events include depression with suicidal ideation, injection site necrosis, and infection which indicate discontinuation of therapy. 

 Interestingly, a recently published study on IFN-*β*1b by Goodin et al. [29] assessed the effect of this medication on survival rate of a randomized cohort of MS patient 21 years following the initiation of the pivotal IFN-*β*1b trial. The study subjects were randomized to receive either the active drug-IFN-*β*1b 250 *μ*g subcutaneously once every other day or placebo. Based on the results of this study, study subjects who were treated with IFN-*β*1b demonstrated a significant decrease in all-cause mortality over the 21-year period compared to placebo recipients (*P* = 0.0173). The authors concluded that early therapy of MS patients with IFN-*β*1b is associated with increased survival in initially untreated patients with RRMS. 

## 5. Interferon-*****β*****1a (Rebif)

This preparation of IFN-*β*1a is manufactured by DNA technology and by the Chinese hamster ovarian cells. Rebif is commercially available at two doses, 22 *μ*g and 44 *μ*g, and is administered subcutaneously three times weekly. IFN-*β*1a (Rebif) was approved in 2002 in the US for treatment of RRMS patients, when its superiority over Avonex (the other approved form of IFN-*β*1a) was demonstrated in the context of the EVIDENCE trial [30]. The EVIDENCE trial was a randomized, multicenter, comparative clinical study that assessed and compared the safety and efficacy of IFN-*β*1a (44 *μ*g subcutaneously three times weekly) over IFN-*β*1a (30 *μ*g intramuscular once weekly) in 677 patients with RRMS. The study evaluators were blinded to treatment and performed neurological and neuroimaging examinations. The primary goal of this trial consisted of the proportion of the patients who were relapse free at 24 weeks, and the major MRI outcome was the number of active lesions per patient per scan at 24 weeks. Based on the results of this comparative trial, after 24 weeks, 74.9% of patients who were treated with IFN-*β*1a 44 *μ*g three times weekly stayed relapse-free compared with 63.3% of those who were treated with IFN-*β*1a 30 *μ*g once weekly. The odds ratio for staying free from relapse was 1.9 (95% CI, 1.3–2.6; *P* = 0.0005) at 24 weeks and 1.5 (95% CI, 1.1 to 2.1; *P* = 0.009) at 48 weeks in favor of IFN-*β*1b 44 *μ*g three times weekly. Treatment of MS patients with IFN-*β*1a 44 *μ*g three times weekly was associated with fewer MR active lesions (*P* < 0.001 at 24 and 48 weeks) compared to those who were treated with IFN-*β*1a 30 *μ*g once weekly. Treatment with high dose high frequency IFN-*β*1a was associated with more injection reactions, more cases of asymptomatic elevation of hepatic enzymes, and a higher incidence of the development of neutralizing antibodies. 

A previous study, the PRISMS clinical trial (prevention of relapses and disability by interferon *β*-1a subcutaneously in multiple sclerosis) [31] assessed the effects of IFN-*β*1a in patients with relapsing-remitting MS. This randomized, double-blind, placebo-controlled trial, which led to the approval of the subcutaneous form of IFN-*β*1a for patients with relapsing-remitting MS, compared IFN-*β*1a (SC) 22 micrograms versus 44 micrograms three times weekly and placebo over a period of two years. The study participants (*N* = 560) were MS patients with an EDSS score between 1.0 and 5.0 and at least two exacerbations in the two years prior to the initiation of the clinical trial. The outcome measures of this clinical trial consisted of relapse rate, progression of disability, and MRI activity. Neurological examination was performed once every three months, with MRI of brain performed twice per year. Analysis was based on intention to treat. After the conclusion of this trial, data was available on 533 of the patients. Analysis of the collected data revealed that the relapse rate was significantly lower at 12 and 24 months with both doses of IFN-*β*1a compared with placebo (mean number per subject 1.82 for 22 *μ*g group and 1.73 for 44 *μ*g versus 2.56 for placebo). The time to first relapse was lengthened by 3 and 5 months in the 22 *μ*g and 44 *μ*g groups, respectively, and the proportion of the relapse-free patients was significantly increased (*P* < 0.05). Treatment with IFN-*β*1a delayed progression in disability, and lowered accumulated disability during the clinical trial. Treatment of MS patients with both doses of IFN-*β*1a had positive impact on accumulation of burden of disease and number of active lesions on brain MR imaging compared to patients receiving placebo. The investigators concluded that treatment of MS patients with IFN-*β*1a was well tolerated and effective in terms of relapse rate, defined disability, and all MR outcome measures in a dose-dependent manner.

Like other beta-interferons, side effects of IFN-*β*1a (Rebif) include flu-like syndrome, injection site pain and redness, hematological and hepatic abnormalities, and depression. Rarely, skin at the injection site becomes infected and necrotic. 

## 6. Interferon-*****β*****1a (Avonex)

Interferon-*β*1a (Avonex) was approved by the FDA in 1996 for the treatment of patients with relapsing form of MS, and similar to Rebif, it is made by Chinese hamster ovarian cells. The FDA approval of this medication followed the results obtained from a clinical trial which was designed by the Multiple Sclerosis Collaborative Research Group (MSCRG) [32–34]. During this phase 3, multicenter, double-blind, placebo-controlled clinical trial, 301 patients with RRMS were randomized to be treated with IFN-*β*1a (30 Mg intramuscularly once weekly) or placebo, for 24 months. The patients' EDSS scores were between 1.0 and 3.5, and each patient had experienced at least two relapses in the three years prior to study initiation. The clinical trial was discontinued earlier than originally designed, and only 57% of patients finished the two-year length of the study. Many conclusions were drawn based on this group of the patients who were treated with two years of IFN-*β*1a and not the whole group which would have included all participants. After 24 months, treatment of MS patients with IFN-*β*1a (Avonex) was associated with an effect on the primary endpoint of the trial, the progression rate of at least 1.0 point on the EDSS compared to placebo. In addition, treatment of MS patients with IFN-*β*1a decreased relapse rate by 18% for the total group and 32% for those who finished the 24-month medication course of the trial. The most frequent side effects of Avonex include flu-like symptoms with headache, fever, chills, fatigue, and vomiting. Other less common side effects consist of depression, suicidal ideation, or deterioration of psychiatric disorders. Elevated liver enzymes have also been reported. 

## 7. Glatiramer Acetate

Glatiramer acetate (GA) (also known as Cop-1 and Copaxone) is a synthetic polymer of random sequences of four naturally occurring amino acids (L-tyrosine, L-glutamate, L-alanine, and L-lysine) and is used as one of the first line disease-modifying agents for the treatment of patient with RRMS. Experimental work has demonstrated that GA suppresses experimental allergic encephalomyelitis (EAE) [35, 36]. GA does not have any biological receptors in the human body, and its exact mechanism of action remains unknown. However, it is believed that GA acts by binding to the major histocompatibility complex class II molecules, competing with the other MS putative antigen(s) such as myelin basic protein for binding to these molecules and to the specific receptors located on the surface of the T lymphocytes [37]. 

 Clinical efficacy of GA in the treatment of patients with MS has been assessed in the context of a number of clinical trials. One initial phase 2 clinical trial of GA in patients with relapsing MS demonstrated a 76% reduction in relapse rate with GA treatment [38]. Another multicenter, placebo-controlled, phase 3 clinical trial showed that treatment of MS patients with GA was associated with reduction of relapse rate by one third, with a significant number of study subjects remaining relapse free [39]. Other clinical trials addressed the effects of GA on MRI parameters [40, 41]. Based on the results of these studies, lesion burden measured by MRI demonstrated improvement in GA-treated MS patients, and GA decreased the frequency of new contrast-enhancing lesions as well as lesion load compared to baseline values. 

 Side effects of GA include self-limited feeling of chest tightness, flushing, anxiety, dyspnea, and palpitation. Flu-like symptoms, which commonly occur after the injection of *β*-interferons, do not happen with GA injections, and treatment of MS with GA is not associated with leucopenia, depression, or elevated hepatic enzymes. 

## 8. Mitoxantrone 

Mitoxantrone (also known as Novantrone) is an antineoplastic drug, structurally related to anthracyclines such as doxorubicin and daunorubicin. Mitoxantrone has immunosuppressive and immunomodulatory features. Mitoxantrone intercalates with DNA, which in turn leads to single- and double stranded breaks. It also suppresses DNA repair by inhibiting the topoisomerase II. Mitoxantrone exerts immunosuppressive effects on proliferating cells such as B and T lymphocytes, decreases secretion of IFN-*γ*, TNF-*α*, and IL-2, and also induces apoptosis of B lymphocytes and monocytes [42–45]. Mitoxantrone possesses dangerous and life-threatening adverse effects including cardiotoxicity, in both cancer and MS patients [46–49], treatment-related acute myelogenous leukemia, and gonadal dysfunction [50–52]. While mitoxantrone is a very effective immunosuppressant with many toxic side effects, its utilization has significantly decreased due to the introduction of other potent and less dangerous medications such as natalizumab.

## 9. Natalizumab 

Natalizumab (Tysabri) is a humanized anti-integrin monoclonal antibody, utilized in treating patients with RRMS [53, 54] and ulcerative colitis [55]. This anti-adhesion monoclonal antibody targets the *α*4-chain of *α*4*β*1 integrin [54, 56], which is also recognized as very late activating antigen-4 (VLA-4) [56]. All leukocytes except for neutrophils express VLA-4 on their surface, which binds to the adhesion molecule, vascular adhesion molecule-1 (VCAM-1), on the surface of activated cerebral endothelial cells. Binding of the activated leukocyte to the inflamed endothelial cells is a crucial step in transendothelial migration of leukocytes to the CNS. The concept of “anti-adhesion therapy” for MS by utilizing a monoclonal antibody stems from the original experiments of Yednock et al. [57] on mice with EAE. These investigators demonstrated that treatment of animals with EAE with anti-VLA-4 monoclonal antibody resulted in a significant decrease in the accumulation of activated leukocytes within the CNS.

 A number of phase 2 clinical trials have evaluated the safety of natalizumab [53, 58], leading to many phase 3 clinical trials. The clinical efficacy of natalizumab for the treatment of MS was assessed during two phase 3 clinical trials: AFFIRM [59] and SENTINEL [60]. During the AFFIRM trial, 924 participants with relapsing MS who had experienced relapses were treated with either natalizumab (300 mg intravenously) or placebo once every 28 days for 24 months [59]. The study participants who were treated with natalizumab had a 68% reduction in clinical relapse rate at 1 year (*P* < 0.001) and a 42% reduction in the rate of disability progression at 24 months (*P* < 0.001). Treatment of MS patients with natalizumab was associated with a 92% reduction of contrast-enhancing lesions (*P* < 0.001), 83% reduction of accumulation of new or enlarging T2-weighted lesions, and a 76% decline in new T1-weighted hypointense lesions (*P* < 0.001).

 During a second phase 3 clinical trial (SENTINEL), 1171 MS patients with relapsing MS who had at least one exacerbation in the year prior to the study while being treated with IFN-*β*1a (IM once weekly) were randomly assigned to be treated with either natalizumab (300 mg IV once every 4 weeks) plus IFN-*β*1a or IFN-*β*1a plus placebo. The results of this clinical trial indicated that combination therapy with IFN-*β*1a and natalizumab was associated with a reduced annualized relapse rate compared to treatment with IFN-*β*1a alone (0.35 versus 0.75; *P* < 0.001) as well as development of fewer new or expanding T2-weighted lesions on brain MRI (*P* < 0.001). At month 24, treatment of MS patients with a combination of IFN-*β*1a and natalizumab was associated with a 24% decrease in the relative risk of sustained disability progression (*P* = 0.02). Currently, natalizumab is utilized for treatment of MS patients and is administered 300 mg IV once every 28 days [61].

 Side effects of natalizumab include headache, fatigue, arthralgia, urinary tract infection, lower respiratory infection, gastroenteritis, vaginitis, diarrhea, and hypersensitivity reactions. An uncommon, but potentially deadly, side effect of treatment of MS patients with natalizumab is the development of an opportunistic infection of oligodendrocytes by JC virus known as progressive multifocal leukoencephalopathy (PML). Clinically, PML manifests with subacute progressive cognitive decline and focal neurological deficits, and it is usually fatal [62, 63]. As of November 1, 2012, there have been 302 confirmed cases of PML in MS patients treated with Tysabri since it became available again in 2006. The risk of developing PML is higher in MS patients who are seropositive for JCV antibodies and those who have previously undergone immunosuppressive therapy with mitoxantrone, methotrexate, or azathioprine Currently, serologic status of the MS patients for JC virus can be determined and this piece of data may assist clinicians with their decision to continue or cease treatment of the MS patients with natalizumab. MS patients who are sero-negative for JCV antibodies should be retested every six months.

It is important to bear in mind that while a definitive cure for MS remains elusive, natalizumab is by far one of the most potent drugs ever developed for treatment of relapsing-remitting MS, and its utilization is associated with prolonged periods of freedom from disease (as evidence by absence of relapses, of disability progression, and of MRI evidence of disease activity) in most of the treated patients.

## 10. Fingolimod

Fingolimod (FTY 720, currently marketed as Gilenya) is an oral sphingosine-1-phosphate (S1P) receptor modulator, approved for treatment of MS in 2010 in North America. This medication is utilized as a second-line drug. S1P receptors are expressed by lymphoid and neural tissues. Sphingosine-based phospholipids are constituents of cell membranes and possess chemoattractive function for the lymphoid cells. Resting T and B lymphocytes express elevated levels of S1P receptor, and lymphocyte exit from the lymph nodes and thymus depends on the activity of this receptor [64–66]. 

 The efficacy of fingolimod in the treatment of MS has been demonstrated in major clinical trials. During one phase 2 clinical trial (with a 2-year extension), its efficacy for treatment of MS was compared to placebo [67]. The TRANSFORMS study was a 12-month, double-blind clinical trial in which 1292 patients with RRMS having a history of at least one relapse were randomized to oral fingolimod (0.5 or 1.25 mg daily) or IFN-*β*1a 30 *μ*g IM once weekly [68]. The primary goal of this study was to assess the annualized relapse rate, and secondary end points included the number of new or enlarged lesions on T2-weighted MR imaging at 12 months as well as progression of disability sustained for at least three months. Of the initial participants, a total of 1153 patients completed the study. The annualized relapse rate was significantly lower in patients in the two arms of the clinical trial who were treated with fingolimod-0.20 in the 1.25 mg group (95% confidence interval; 0.16–0.26) and 0.16 in the 5 mg group of the study (95% confidence interval; 0.12–0.21) compared to the group treated with IFN-*β*1a (0.33; 95% confidence interval, 0.26–0.42; *P* < 0.001). 

A rare but significant issue associated with the use of fingolimod, is the development of the herpes zoster infection and its associated neurological complications. Varicella-zoster virus (VZV) is a neurotrophic and exclusively human virus causing chicken pox (varicella). Once contracted, the virus remains, as a latent agent, within the ganglionic neurons along the neuroaxis. Based on available study data, two cases of fatal herpes virus family infections occurred. Results indicated that one patient with herpes simplex virus encephalitis died during the trial, and another patient with primary disseminated VZV infection died as well. These herpes-related fatal outcomes occurred during the clinical trial of fingolimod in MS patients who were treated with a higher dose of the medication [68]. Therefore, patients who are not immunized against VZV should be vaccinated prior to initiation of therapy with fingolimod. 

Other practical considerations with clinical utilization of fingolimod, particularly following administration of the first dose, are bradycardia, bradyarrhythmias, and mild reduction of forced expiratory volume in 1 second. Such side effects stem from the fact that in addition to its presence on the lymphocytes, the sphingosine-phosphate receptor is also expressed on other tissues such as atrial myocytes. Due to this effect, a 6-hour observation period is advised once the first dose of fingolimod is administered. 

## 11. Alemtuzumab

Alemtuzumab (also known as Campath-1H) is a humanized monoclonal antibody which targets cell surface molecule CD52-a glycoprotein antigen expressed on the surface of mature lymphocytes and monocytes. CD52 is also expressed by other cells such as thymocytes and macrophages. However, stem hematopoietic cells, plasma cells, and platelets do not express the CD52 antigen [69]. Currently, alemtuzumab is approved by the FDA for treatment of B lymphocyte chronic lymphocytic leukemia. Alemtuzumab depletes cells which carry CD52 via different routes, including complement-mediated lysis, antibody-dependent cell toxicity, and apoptosis. It has also been demonstrated that alemtuzumab induces production of neurotrophic factors by the reconstituted autoreactive T lymphocytes [70]. One line of reasoning for utilization of alemtuzumab for treatment of MS rests on the concept that with profound depletion of lymphocytes by this monoclonal antibody, the reconstituted pool of T lymphocytes will be devoid of autoreactive clones of T lymphocytes which promote neuroinflammation in the context of MS [71], which in turn reduces CNS inflammatory damage.

 Efficacy of alemtuzumab for the treatment of MS has been assessed through a number of clinical trials. CAMMS223, a 36-month, phase 2, rater-blinded trial included 334 subjects with RRMS whose disease duration was ≤3 years. The study participants were randomized to annual intravenous cycles of alemtuzumab (12 or 24 mg/day) versus IFN-*β*1a (44 *μ*g subcutaneously three times weekly) for the length of the clinical trial [72]. Treatment with alemtuzumab was associated with a significant reduction of annualized relapse rate compared to IFN-*β*1a (0.10 versus 0.36, *P* < 0.001) as well as significantly decreased T2-weighted lesion burden than IFN-*β*1a (*P* = 0.005) [72]. Patients who were treated with alemtuzumab experienced a significantly lower rate of sustained disability accumulation versus IFN-*β*1a (9.0 versus 26.2%, *P* < 0.001), as evidenced by improvements of the EDSS score. Based on the results of one planned post hoc analysis, more patients who were randomly treated with alemtuzumab had achieved sustained decrease in disability compared to those who were treated with IFN-*β*1a (hazard ratio = 2.61, 95% CI = 1.5–4.4; *P* = 0.0004) [73]. 

 Recently, the results of a phase 3 clinical trial of alemtuzumab in treatment of patients with RRMS were published [74]. The first study assessed the efficacy of alemtuzumab versus IFN-*β*1a (Rebif) for patients with RRMS. During this randomized controlled 2-year trial. 187 of 195 participants who were randomized to IFN-*β*1a and 376 of 386 patients allocated to alemtuzumab were included in the primary analyses. Based on the results of this study, 75 (40%) patients in the IFN-*β*1a group experienced relapses (122 events), while only 82 (22%) patients in the alemtuzumab group relapsed (119 events; rate ratio 0*·*45 [95% CI 0*·*32–0*·*63]; *P* < 0.0001), corresponding to a 54*·*9% improvement with alemtuzumab. The authors concluded that the efficacy and safety profile of alemtuzumab in treatment of treatment-naïve MS patients supports its utilization in these patients.

## 12. Dimethyl Fumarate

Dimethyl fumarate (BG00012), an ester derivative of fumaric acid, possesses immunomodulatory properties and is a potential oral treatment of MS. BG-12 has shown beneficial effects in treatment of EAE and may reduce transendothelial migration of activated leukocytes through the blood brain barrier along with neuroprotective effects via activation of antioxidative pathways [75, 76].

 The efficacy of BG-12 for treatment of MS was assessed during DEFINE trial. This clinical trial was a 2-year, phase 3, randomized, double-blind, placebo-controlled, dose-comparison study of BG-12 in 1234 patients during which study subjects were randomized to two different doses of BG-12 (either 240 mg PO BID or 240 mg PO TID) or to placebo. Results of this clinical trial demonstrated the superior effect of both doses of BG-12 over placebo in significant reduction in the proportion of patients who relapsed at 2 years compared to placebo (*P* < 0.0001). Both doses of BG-12 were superior to placebo in reducing the annual relapse rate, the number of new or newly enlarging T2-weighted hyperintense lesions, and confirmed disability progression [77]. Based on the results of the DEFINE study, BG-12 had a safety profile comparable to placebo.

 One of the largest published studies on BG-12 and relapsing-remitting MS stems from phase 2, randomized, double-blind, placebo-controlled, dose-ranging study which included 257 participants with relapsing-remitting MS. The study participants were randomly treated with oral placebo versus BG-12 120 mg, 360 mg, or 720 mg orally daily for 24 weeks. In the 24-weeks extension phase of this trial, study participants who were treated with placebo were switched to BG-12 720 mg orally daily. The primary outcome of this study was the total number of new contrast-enhancing lesions on brain MR scans at weeks 12, 16, and 24. Other outcomes included cumulative number of new contrast-enhancing lesions, new T1-weighted hypointense lesions at 24 weeks and annualized relapse rate. According to the results of this clinical trial, treatment of MS patients with BG-12 240 mg orally three times daily was associated with 69% decrease in the mean total number of new contrast-enhancing lesions compared to the placebo group (1.4 versus 4.5, *P* < 0.0001). Treatment with BG-12 was also associated with a decrease in the number of new or expanding T2-hyperintense lesions (*P* = 0.0006) and new T1-weighted hypointense lesions (*P* = 0.014) compared to placebo. In addition, treatment of MS patients with BG-12 decreased the annualized relapse rate by 32%. Adverse events of treatment with BG-12 included abdominal pain, flushing, and hot flush. Dose-related events in recipients of BG-12 consisted of headache, fatigue, and feeling hot [78]. 

Two recently published papers in the New England Journal of Medicine have reported the efficacy of BG-12 in treatment multiple sclerosis [79, 80]. The first report by Fox et al. [79] (CONFIRM study) presents the results of a placebo-controlled phase 3 clinical trial of BG-12 or glatiramer acetate in patients with relapsing-remitting MS. During this trial, the study participants were randomized to BG-12 at a dose of 240 mg orally two or three times daily, or placebo. The study also included glatiramer acetate as a comparator treatment arm. The primary endpoint of the CONFIRM clinical trial was the annualized relapse rate during a period of 24 months. This clinical trial did not aim to assess the superiority or lack of superiority of oral BG-12 against glatiramer acetate. Based on the results obtained from this clinical trial, after 24 months, the annualized relapse rate was lower in MS patients treated with BG-12 twice every day (0.22), three times daily (0.20), and glatiramer acetate (0.29) compared to placebo (0.40) (relative decreases: two times daily BG-12. 44%, *P* < 0.001; three times daily BG-12, 51%, *P* < 0.001; glatiramer acetate, 29%, *P* = 0.01). Compared to the placebo, treatments with BG-12 twice daily and BG-12 three times daily as well as glatiramer acetate were associated with a significant decrease in the numbers of new or expanding T2-weighted hyperintense lesions (all *P* < 0.001) and new T1-weighted hypointense lesions (*P* < 0.001, *P* < 0.001, and *P* < 0.002, resp.). Adverse events were more common in patients treated with active BG-12 or glatiramer acetate and consisted of flushing and gastrointestinal events (BG-12) or injection site reactions with glatiramer acetate. The adverse events did not include any opportunistic infections or malignant cancers. Treatment with BG-12 is associated with low lymphocyte counts. The investigators concluded that treatment of patients with relapsing-remitting MS with BG-12 and glatiramer acetate caused significant reduction in annualized relapse rate and improved neuroradiologic findings compared to the placebo.

The second paper published by Gold et al. [80] presents the results from a phase 3, placebo-controlled trial of oral BG-12 for treatment of patients with relapsing MS. The investigators executed a randomized, double-blind, placebo-controlled study of oral BG-12 in patients with MS. Study participants were randomly assigned to treatment with oral BG-12 m at a dose of 240 mg twice every day, BG-12 240 mg three times daily, or placebo (DEFINE study). The primary endpoint of the study consisted of the proportion of patients who experienced one relapse within two years. A number of other aims included annualize relapse rate, the time towards confirmed progression of disability, and neuroimaging parameters. Based on the results of this clinical trial, the MS patients treated with BG-12 experienced significantly less relapses (noted in both BG-12 dosing groups) compared to patients receiving placebo (27% with BG-12 two times daily and 26% with BG-12 three times daily compared to 46% with placebo, *P* < 0.001). The annualized relapse rate after 24 months was 0.17 in the BG-12 twice daily group, 0.19 in the BG-12 three times daily group, and 0.36 in the placebo-treated arm of the study (*P* < 0.001 for the comparison of each BG-12 regimen with placebo). According to the results of this clinical trial, the estimated proportion of patients with confirmed progression of disability was 16% in the BG-12 twice daily arm, 18% in the BG-12 three times daily group, and 27% in the placebo-treated arm, with significant relative risk reductions of 38% with BG-12 two times daily (*P* = 0.005) and 34% with BG-12 three times daily (*P* = 0.01). Treatment of MS patients also significantly decreased the quantity of contrast-enhancing lesions and new or expanding T2-weighted hyperintense lesion on brain MR (*P* < 0.001 for the comparison of BG-12 regimen versus placebo). Treatment of MS patients with BG-12 was associated with adverse events such as abdominal pain, nausea, diarrhea, lowered lymphocyte counts, and increased hepatic transferases levels. The authors concluded that treatment of relapsing MS patients with BG-12 (both dosing regimens) significantly decreased the number of relapses, the annualize relapse rate, the rate of disability deterioration, and the number of MRI lesions.

## 13. Teriflunomide

Teriflunomide (Aubagio) (a derivative of leflunomide) is an oral drug which binds to dihydro-orotate dehydrogenase (DHODH) and reversibly inhibits it. DHODH is a mitochondrial membrane protein which is essential for pyrimidine synthesis [81, 82]. It is believed that such suppression of pyrimidine synthesis in rapidly proliferating cells, such as T and B lymphocytes, is responsible for the immunomodulatory effects of teriflunomide [83]. One preliminary phase 2 proof-of-concept, randomized, double-blind, placebo-controlled clinical trial assessed the safety and efficacy of teriflunomide in MS patients with relapses [84]. Study participants were randomized to be treated with either placebo, teriflunomide 7 mg/daily, or teriflunomide 14 mg/daily. According to the results of this study, treatment of MS patients with teriflunomide was associated with a reduction of combined unique active lesions per MRI scan during the 36-week treatment phase. Teriflunomide was well-tolerated by patients with relapsing MS. Another randomized clinical trial of oral teriflunomide for patients with relapsing MS assessed the annualized relapse rate and confirmed progression of disability in these patients [85] (TEMSO study). During this clinical trial, 1088 MS patients, 18 to 55 years of age, with an EDSS score of 0.0 to 5.5 and at least one relapse in the year or two relapses in the two years prior to study initiation, were randomized to either placebo, teriflunomide 7 mg/daily, or teriflunomide 14 mg/daily for 108 weeks in a 1 : 1 : 1 pattern. Compared to placebo, treatment of MS patients with teriflunomide was associated with 31.2% and 31.5% reduction in annualized relapse rate in the 7 mg/daily and 14 mg/daily treatment groups, respectively (*P* < 0.001 for both comparisons with placebo). In addition, treatment of MS patients with teriflunomide (both doses) had a positive impact on MRI outcomes. Significant side effects occurring in patients treated with teriflunomide consisted of diarrhea, nausea, hair thinning, and mildly increased hepatic enzymes. 

## 14. Laquinimod

Laquinimod, a derivative of linomide, is an immunomodulatory agent which is used as a once-daily oral drug for treatment of MS. While the exact therapeutic mechanism(s) of action of laquinimod in MS remains unknown, it has been demonstrated that laquinimod promotes anti-inflammatory cytokine profile in human peripheral blood mononuclear cells [86]. In EAE model of MS, laquinimod decreased inflammation, demyelination, and axonal injury [87–89]. 

 Laquinimod has been assessed for treatment of MS in the context of one phase 3 clinical trial. During a 2-year, phase 3, randomized, double-blind, placebo-controlled clinical trial (ALLEGRO), 1106 patients with relapsing-remitting MS were randomized to treatment with 0.6 mg laquinimod once daily versus placebo. The primary end point consisted of the annualized relapse rate during the 24-month study, while the secondary end points were confirmed disability progression and the cumulative number of contrast-enhancing lesions and new or enlarging lesions on T2-weighted MR sequence [90]. Treatment with laquinimod was associated with a modest decrease in annualized relapse rate versus placebo (0.30 ± 0.02 versus 0.39 ± 0.03, *P* = 0.002) along with a decrease in the risk of confirmed disability progression (11% versus 15.7%, hazard ratio. 0.64, confidence interval 95%; *P* = 0.01). The mean cumulative numbers of contrast-enhancing lesions and new or enlarging T2-weighted lesions were less in patients who received laquinimod. In addition, treatment of MS patients with laquinimod was associated with a 33% decrease in progression of brain atrophy compared to placebo (*P* < 0.0001).

## 15. Rituximab

Rituximab (Rituxan) is a chimeric (human/mouse) monoclonal antibody with IgG1 heavy-chain and kappa light chain constant region sequences and mouse variable region sequences, which depletes CD20^+^ B lymphocytes via cell-mediated and complement-dependent cytotoxic effects and promotes apoptosis of these cells [91]. In 1997, the FDA approved use of rituximab for the treatment of relapsing or refractory cases of low grade or follicular CD20^+^ B lymphocyte non-Hodgkin lymphomas. CD20 antigen is a 35 kDa transmembrane protein which is expressed by majority of B lymphocytes in patients with non-Hodgkin lymphomas. While normal B lymphocytes and its precursors express this antigen, plasma cells, T lymphocytes, and hematopoietic stem cells do not possess CD20 antigen. As a B lymphocyte depleting drug, administration of rituximab leads to rapid abolition of CD20^+^ B lymphocytes in the peripheral circulation [92, 93]. One phase 2 clinical trial assessed efficacy of rituximab in patient with relapsing-remitting MS, and results of this study indicated that treatment of MS with rituximab was associated with decline of contrast-enhancing lesions versus placebo (−91%, *P* < 0.001) as well as significant reduction in risk for relapse (20.3 versus 40.0%, *P* = 0.04) [94]. 

## 16. Daclizumab

Daclizumab (also recognized as Zenapax) is a humanized monoclonal antibody targeting the *α*-subunit of IL-2 receptor CD25 on activated T lymphocytes. IL-2 which is a T lymphocyte growth factor has a significant task in beginning the proliferation or clonal expansion of antigen-stimulated T lymphocytes [95]. Therefore, blocking the CD25 on the activated T lymphocytes downregulates proliferation of B and T lymphocytes via reducing the secretion of pro-inflammatory cytokines [96, 97]. Currently, daclizumab is utilized along with other immunosuppressive drugs to circumvent renal graft rejection [98]. Clinical studies of daclizumab in MS patients indicate that its clinical efficacy is exerted via production of CD56^+^ natural killer cells with regulatory function [99]. 

Daclizumab was assessed for treatment of MS in the context of two clinical trials. During the first multicenter, placebo-controlled trial (SELECT), a cohort of 600 patients with MS was randomized in a 1 : 1 : 1 ratio to be treated with daclizumab 150 mg subcutaneously every 4 weeks, daclizumab 300 mg subcutaneously every 4 weeks, or placebo [100]. The primary outcome of this study consisted of its effect on annual relapse rate at 12 months. At one year, the annual relapse rate for placebo was 0.46, while it was 0.21 for daclizumab 300 mg group and 0.23 mg for daclizumab 150 mg group, respectively (*P*, 0.001). A second phase II clinical trial (CHOICE), which included 230 patients with active relapsing-remitting MS, already being treated with IFN-*β*, assessed the efficacy of daclizumab for the treatment of MS. Study participants were randomly assigned to be treated with add-on subcutaneous daclizumab 2 mg/kg once every 2 weeks, subcutaneous daclizumab 1 mg/kg once every 4 weeks, or placebo for a period of 24 weeks. Of these, 46% of patients were on subcutaneous, 30% of IFN-*β*1a intramuscular, and 24% on IFN-*β*1b subcutaneous. The primary endpoint of this study was the total number of new or enlarged contrast-enhancing lesions which were detected between weeks 8 and 24. Based on the results of this clinical trial, both add-on daclizumab groups had lower number of new or enlarged contrast-enhancing lesions (1.32 for high dose daclizumab and 3.58 for low dose daclizumab) compared to the group treated with IFN-*β* and placebo (4.75) (*P* = 0.004).

Based on the safety information obtained from the CHOICE study, infection rates were similar across all treatment groups. However, the incidence of cutaneous adverse events was higher in the combined daclizumab groups compared to the placebo group. A higher rate of grade-3 or grade-4 infections happened in patients who were treated with daclizumab compared to the placebo group. Patients who were treated with daclizumab did not develop opportunistic infections, and all infection resolved with treatment [101].

## 17. Cladribine

Cladribine is a potent immunosuppressive agent. Its active metabolite suppresses DNA synthesis and repair, which in turn results in apoptosis of lymphocytes [102]. During a large clinical trial (CLARITY), cladribine was assessed for the treatment of patients with relapsing-remitting MS. While cladribine was found to be effective for treatment of these patients, certain concerns regarding its prolonged immunosuppressive effects as well as increased risk for cancer caused the withdrawal of applications for marketing approval in Europe and cessation of further follow-up development in the United States. 

## 18. Concluding Remarks

Prior to 1993, there were no effective treatments for MS, and most patients developed significant disability and disease progression a few years from disease onset. However, currently, there are at least 8 FDA-approved treatments for MS, and much effort and emphasis are placed on development of safer and orally available medications for treatment of MS. While we are still far from finding a cure for MS, small but persistent steps are being taken in that direction, and the future looks bright for MS patients. 
